# Development of a Novel Low-Cost Emergency Airway Simulator to Improve Access to Clinical Training in Resource-Limited Paramedicine

**DOI:** 10.7759/cureus.103035

**Published:** 2026-02-05

**Authors:** Brian F Quach, Yash Verma, Gabriel Q Borland, Andrew Eyre

**Affiliations:** 1 Medicine, Frank H. Netter MD School of Medicine, North Haven, USA; 2 Medicine, Drexel University College of Medicine, Philadelphia, USA; 3 Science, University of Massachusetts Boston, Boston, USA; 4 Emergency Medicine, Brigham & Women’s Hospital, Boston, USA

**Keywords:** airway mangement, basic and advanced training in anesthesiology, laryngoscopy and endotracheal intubation, low- and middle-income country, low-resource setting, paramedic emergency medical services, procedural skills training, simulation in medical education, surgical cricothyrotomy, teaching in emergency medicine

## Abstract

Effective airway management is a cornerstone of prehospital emergency care, often determining the outcome of critically ill or injured patients. However, prior studies have identified several possible risk factors that hinder the performance of advanced airway management procedures in the field, such as endotracheal intubation (ETI) and cricothyrotomy. These risk factors may include facial trauma, airway obstruction, trismus, or confined rescue environments. Medical simulation is an educational modality for emergency medical services (EMS) providers to build their practical skills in a controlled, psychologically safe environment. However, due to economic and supply chain issues, it may be challenging for EMS providers working in low- and middle-income countries (LMICs) to obtain high-quality commercial training products to practice these procedures. To address this limitation, we designed and built an innovative, cost-effective task trainer using readily available materials that offers immediate feedback on the correct placement of advanced airway devices.

## Introduction

In the emergency medical services (EMS) setting, the ability to establish an advanced airway is among the most critical life-sustaining skills clinicians must possess when managing patients with inadequate oxygenation [1-5]. Within the globally recognized Airway, Breathing, Circulation (ABC) trauma response framework, securing a patent airway is universally identified as a top priority, and failure to do so is strongly associated with adverse outcomes in some patients [6,7]. While supraglottic airway (SGA) devices offer a useful and often simpler alternative for emergency responders, endotracheal intubation (ETI) remains the gold standard for securing the airway in the most critical cases [8-11]. However, despite its clinical advantages, ETI is a technically challenging skill that requires a solid understanding of airway anatomy and has a significant learning curve. Prior research has shown that failure rates can be as high as 23% in EMS systems relying on non-physicians, compared to just 1% in physician-manned EMS systems [12-15]. These data highlight the need for more rigorous training and skill maintenance among EMS providers. When other airway management methods fail, such as in cases of severe bleeding or airway obstruction secondary to facial trauma, cricothyrotomy is widely recognized as a last-resort option for establishing an airway [16]. Since the widespread implementation of supraglottic airway (SGA) devices, the incidence of cricothyrotomy has decreased. It is an invasive, technically demanding procedure that carries a high risk of long-term complications if executed incorrectly [17,18]. Despite its rarity, available evidence suggests that cricothyrotomy has a higher success rate than ETI, with failure rates not exceeding 16.9% in non-physician EMS systems, compared to 6% in physician-manned systems [15,18,19]. However, due to its lower incidence, paramedics may have less real-world experience with the procedure, which may make it more challenging to maintain proficiency. 

For emergency medical technicians (EMTs) and paramedics, field experience alone may be insufficient to maintain the technical expertise required for procedures such as ETI and cricothyrotomy, as opportunities for practice may be limited or occur under extremely stressful conditions where failure can compromise patient safety. Medical simulation has emerged as a valuable educational tool to address this challenge, providing a psychologically safe environment where providers can practice technical skills and learn from mistakes without harming patients. Task trainers are physical simulation models designed to develop and maintain proficiency in specific technical skills. Unfortunately, commercial airway simulation task trainers are often prohibitively expensive, often exceeding USD 2,000, making them inaccessible to many EMS systems, particularly those in resource-limited settings. To address these barriers, we developed a high-fidelity advanced airway task trainer for USD 107 using readily available materials that preserves the educational value of commercial models while making training more accessible to providers who practice in these areas.

## Technical report

This article aims to describe the design and development of a low-cost, high-fidelity task trainer for practicing advanced airway procedures, including ETI and cricothyrotomy. Our objective is to improve access to simulation education for rural and resource-limited EMS systems without compromising educational value, ultimately hoping to enhance procedural confidence and reduce prehospital airway management failure rates.

The construction of our advanced airway trainer involved a stepwise assembly of multiple components designed to replicate key anatomical structures and provide realistic tactile feedback (see Appendix A). A comprehensive list of materials and their associated costs is presented in Table 1.

**Table 1:** Materials and components used to create the device Material costs may vary over time due to changes in retail pricing, manufacturers, or quantity purchased. Costs are reflective of the amount the team paid for products from North American retailers, including Walmart (Bentonville, Arkansas, USA) and Home Depot (Atlanta, Georgia, USA). The specific brands listed above are what we used for our prototype and are not essential to replicate the design. To further reduce expenses, we repurposed leftover ballistic gel from a commercial block that was purchased for a previous project. For those seeking a more economical alternative to purchasing a pre-made gel block, a comparable block can be created by mixing water with ballistic gel powder. Ballistics gel previously used in another project was incorporated into this work [20]. ASIN is provided as a universal reference code for products used in the construction of this trainer.

Model components	Cost (in United States Dollars (USD))	Quantity	Amazon Standard Identification Number (ASIN)
SimBowel Segment	29.98	1	B09FC5DD27
Foam ball	1.99	1	B09R7SYMT8
Simulated teeth	5.99	1	B097SRJLWX
Simulated tongue	3.00 each	2	B0DM6GSFNK
Heavy-duty all-weather adhesive tape	11.98	1	B07GRJ8L55
Lubricating jelly	0.13 each	4	B00ZVU6JVI
1¼ in. x 2 ft. Poly Vinyl Chloride (PVC) Sch. 40 pipe	4.37	1	B0047EAOY2
1¼ in. PVC Sch. 40 Tee	3.37	1	B075VBJCKB
1 in. Sch. 40 45° elbow	1.60 each	2	B0B52YFLX7
PTFE plumbers tape	0.98	1	B0CGY4RCPW
Heavy-duty clear PVC cement	9.98	1	B000HE5EBE
Zip ties	0.02 each	10	B0BXF7JBBZ
Rubber bands	0.01 each	10	B003U6N3GY
Balloons	0.97	3	B0D4PF6LX2
Ballistics gel block	13.00	1/10 of block measuring 16 inch length x 6 inch width x 6 inch height	B0B9YNSFYP
2x4 - 2 ft. pre-cut lumber	1.75	1	B0FBHZKQ9N
¾ in. x 11¾ in. x 2 ft. white melamine board	8.98	1	B0CXNDXRXH
8-inch contractor wood shims	0.15	10	B084GZ28RK
2 in. wood screws (pack of 12)	0.21 each	12	B07QNZNS1N
Total Cost	USD 107.33	N/A	N/A

Clinical supervision for model making was provided by a board-certified emergency medicine physician and nationally registered paramedic, both with substantial clinical experience and familiarity in performing emergency airway procedures.

**Figure 1:**
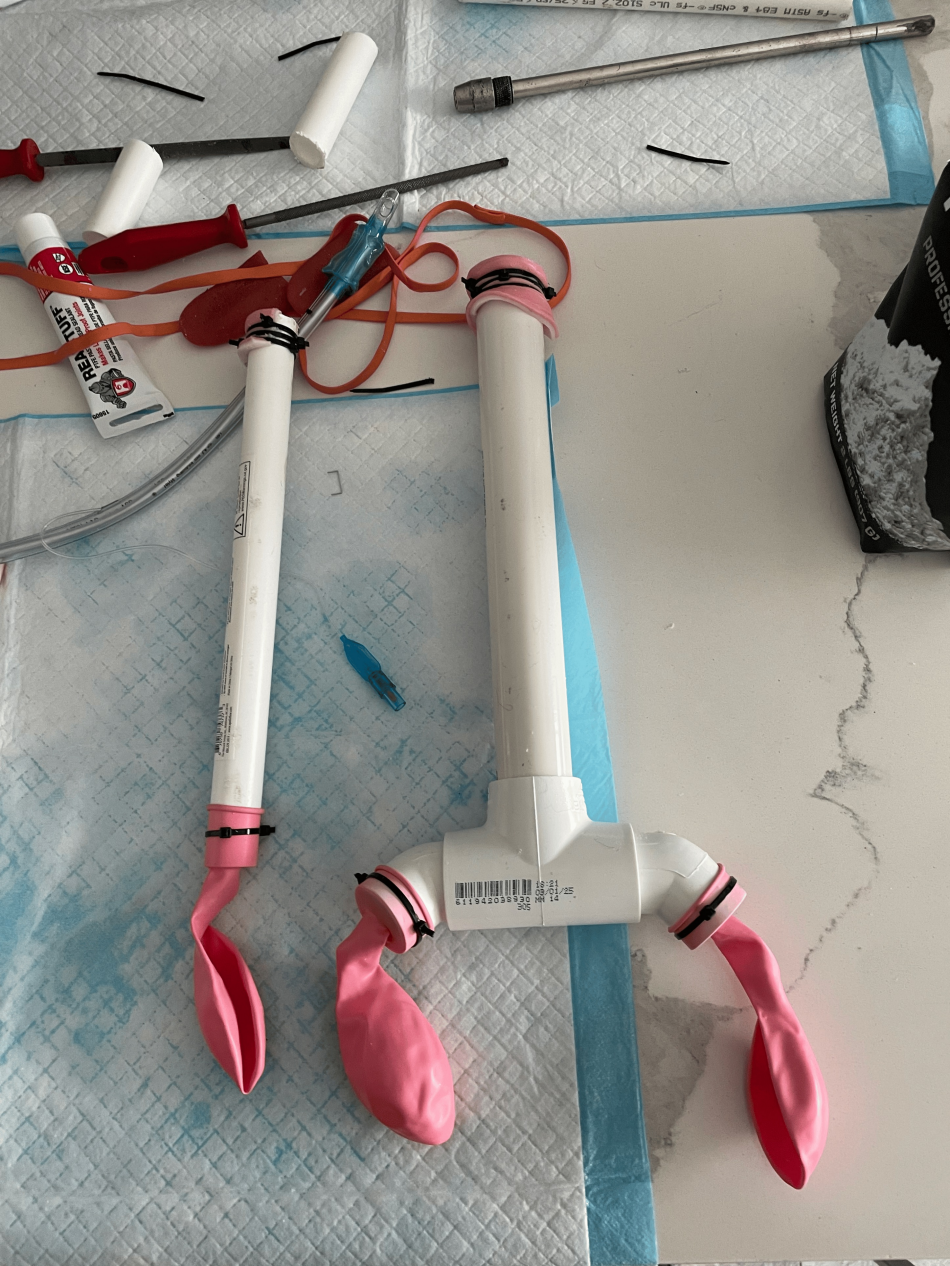
Prepared trachea and esophagus with a simulated bowel lining and balloon This figure shows the pre-assembled trachea and esophagus with a balloon feedback mechanism. The simulated trachea was later shortened to enhance anatomical realism.

The head was constructed from a six-inch foam ball, with openings for the mouth, trachea, and esophagus created using a power drill. Simulated teeth and tongue were affixed using heavy-duty adhesive tape to form a realistic oral cavity. Sections of simulated bowel tissue were used to line the trachea and esophagus, creating a texture that simulates tracheal rings and smooth muscle, respectively, for ETI practice. The opposite end was fitted with a balloon that provided immediate visual feedback during tube placement and bag-valve ventilation (Figure 1).

**Figure 2:**
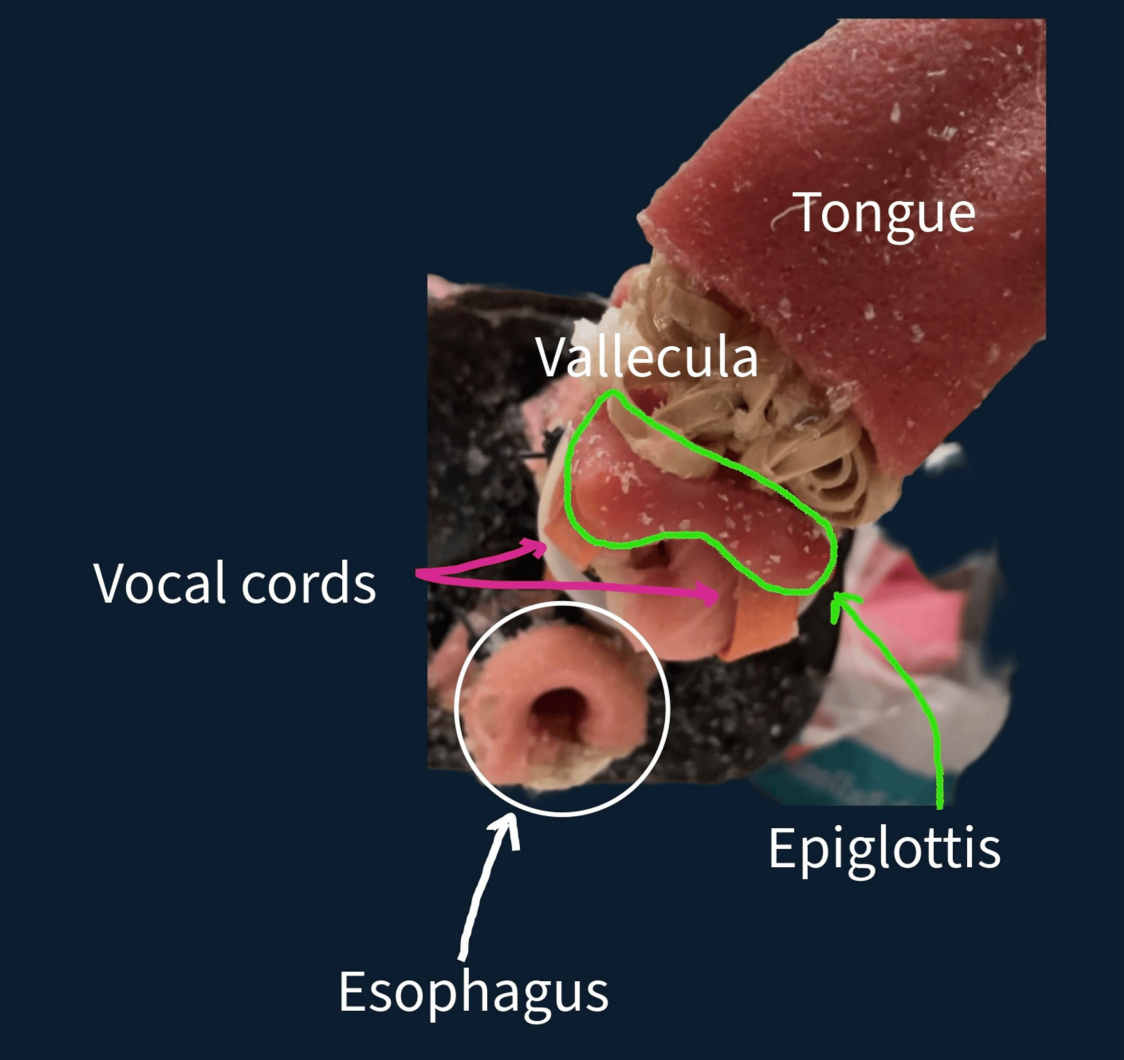
Annotated top-down view highlighting the tracheal components The panel is a top-down view of the completed upper airway structures, including the simulated tongue, vocal cords, epiglottis, and vallecula, arranged to replicate the key anatomical landmarks encountered during laryngoscopy. The landmarks are labeled accordingly.

The trachea was further modified by drilling an opening at the anatomical position of the cricothyroid membrane, and sized to accommodate a cuffed tracheostomy tube. This opening was covered with heavy-duty adhesive tape to replicate the membrane’s resistance. To simulate the appearance of the vocal cords, strips of a thick rubber band were stretched across the tracheal lumen and anchored in place with zip ties. A separate piece of simulated tongue material was trimmed to form an epiglottis and secured above the tracheal lumen, also using zip ties. Finally, a vallecula was created by adhering a compact ball of folded rubber bands between the primary tongue and the newly placed epiglottis with contact adhesive, creating realistic laryngoscopic landmarks (Figure 2).

Once the airway assembly was complete, lubricating jelly was applied to the trachea and esophagus to ensure smooth passage of the tube and reduce friction.

**Figure 3:**
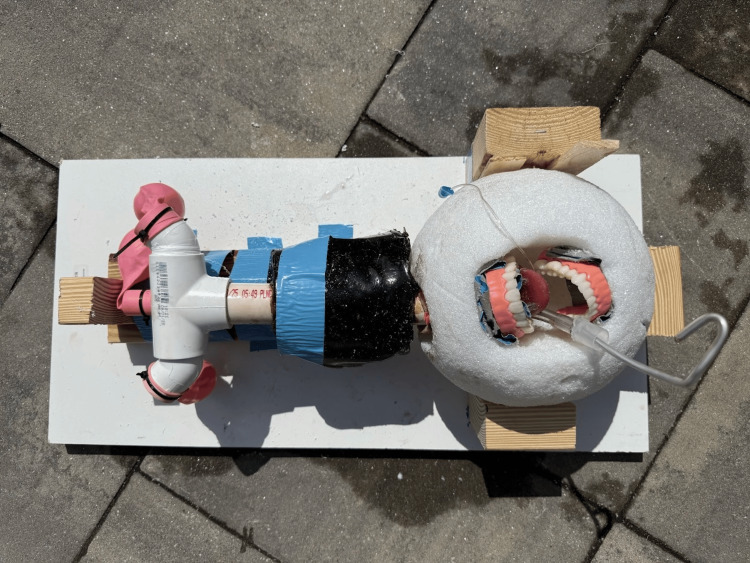
Final assembly of the low-cost advanced airway task trainer The low-cost advanced airway task trainer in its final assembled state with endotracheal tube secured in place, featuring a melamine platform and lumber supports to ensure consistent positioning and secure handling.

The neck was cast by melting ballistic gel in a slow cooker and pouring it into a cylindrical plastic container, where the preassembled trachea and esophagus were positioned to orient the cricothyroid membrane anteriorly. The gel was allowed to solidify completely before the container was removed. The final assembly was mounted onto a custom platform constructed from a melamine wood shelf and 2x4 lumber supports, which held the head securely during repeated practice sessions, as shown in Figure 3.

Additional stabilization was achieved using shims and heavy-duty tape as needed. 

Results

**Figure 4:**
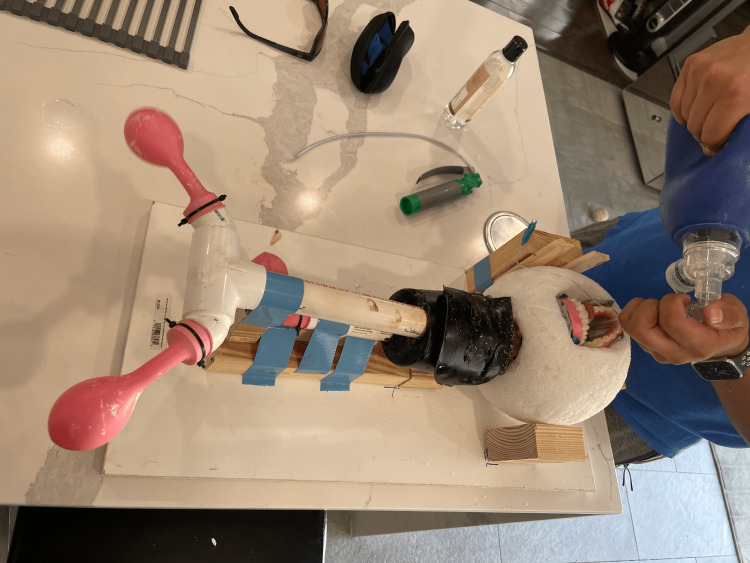
Visual feedback of the airway ventilation with balloons

We developed a low-cost advanced airway task trainer using readily available materials with a total construction cost of USD 107.33. While the material cost was minimal, additional considerations included approximately six hours of assembly time and basic construction expertise, representing a modest trade-off for the substantially reduced financial expense. The completed model accurately replicates key upper airway landmarks, including the tongue, epiglottis, vallecula, vocal cords, and cricothyroid membrane, enabling learners to practice various advanced airway procedures, such as ETI and cricothyrotomy, with realistic tactile feedback. The integrated feedback mechanism, achieved through balloon inflation, enables immediate confirmation of proper tube placement and ventilation (Figure 4).

**Figure 5:**
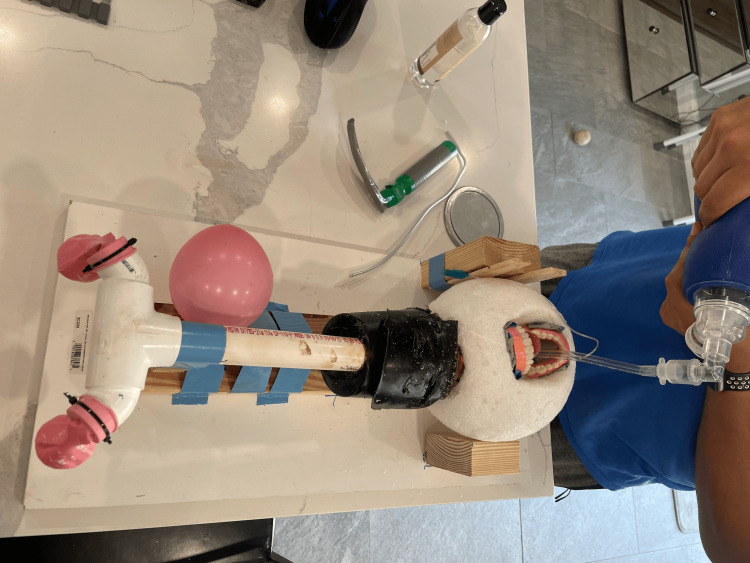
Visual feedback simulating incorrect endotracheal tube placement during the procedure

If placed incorrectly, the balloon attached to the simulated esophagus will inflate (Figure 5).

The platform's stability supports repeated use without compromising durability, while its modular design allows for the easy replacement of worn components. This trainer offers an accessible, high-value alternative to commercial models, providing an effective tool for developing and practicing procedural skills among resource-limited EMS providers.

## Discussion

Advanced airway management procedures, such as ETI and cricothyrotomy, remain critical skills for EMS providers, yet failure rates among non-physician prehospital providers are notably higher than those reported in physician-manned EMS systems [12-15,18,19]. To help address this gap, we constructed a high-quality, low-cost training tool designed to improve the proficiency of advanced airway procedures performed by EMS workers, providing accessible and realistic practice opportunities. While our primary target is EMS providers and first responders in resource-limited areas, this model can be used to support the clinical education of other non-EMS clinicians, such as community health workers, nurses, and physicians practicing in these settings. 

The development of this low-cost advanced airway task trainer demonstrates that practical and realistic simulation tools do not have to be prohibitively expensive to retain educational value. Commercial airway trainers can cost several thousand dollars, creating barriers for many smaller EMS systems, rural educational programs, and resource-limited healthcare systems. By using repurposed, readily available materials, we developed an advanced airway task trainer with a net cost of USD 107.33, while still preserving key educational features such as an anatomically accurate airway, realistic soft tissue resistance, and compatibility with standard airway equipment. Although primarily designed for ETI and cricothyrotomy training, this versatile model can also support the practice of several alternative rescue techniques, including the placement of SGA devices, airway foreign body removal, bag-valve-mask (BVM) ventilation, surgical and needle cricothyrotomy, retrograde intubation, and oropharyngeal airway (OPA) insertion. 

Future studies and development

In a future study, we intend to pilot this model as part of a continuing education program for paramedics and advanced EMTs at a private EMS agency in the northeastern United States. We plan to evaluate the educational efficacy of this trainer by measuring learner confidence, procedural success rates, and long-term skill retention when training on this model. Evaluations will be measured using Likert scores and short answer responses.

Potential refinements could also enhance its utility, such as modular trachea upgrades or inflated tongues that can simulate difficult-airway scenarios. Although we have not yet measured its impact on clinical performance, we hope that expanding access to this low-cost trainer could significantly benefit resource-limited EMS systems and educational programs by contributing to improved success rates of advanced airway procedures.

Limitations of the simulator 

**Figure 6:**
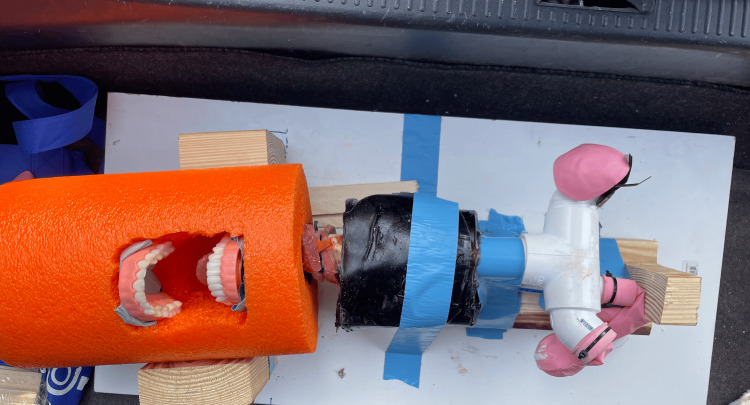
Alternative airway trainer made from a section of a foam roller A cut section of an 18-inch high-density foam roller is used to simulate the head of the airway trainer, offering improved durability and shape retention compared to other foam materials.

While this trainer offers several advantages, it does have limitations. First, the spherical shape of the foam head prevents certain maneuvers, such as jaw thrust, which makes practicing BVM ventilation more challenging. This issue is compounded when the openings for the trachea and esophagus are drilled too large, as air can escape through the gaps instead of reaching the balloons designed to provide immediate feedback. A similar limitation applies to the placement of the SGA devices, where a loose seal around the airway structures can result in ineffective ventilation feedback. To address these issues, we recommend drilling smaller, precise openings that allow a snug fit of the trachea and esophagus into the foam head. Applying a flexible sealant such as silicone caulk, hot glue, or expanding spray foam sealant around the junction can further prevent air leakage while maintaining durability. Additionally, the placement of plastic face masks with embedded holes over these models can serve as an adjunct for practicing OPA insertion and BVM ventilation as the hole could serve as a tight seal for more effective air passage, potentially enhancing the realism of our model. Another limitation is the gradual degradation of the foam material used in this project, particularly when exposed to adverse environmental conditions, such as heat or consistent mishandling. Replacing the standard foam ball with a section of high-density foam, such as that used in foam rollers (Figure 6), may increase longevity and maintain structural integrity over time.

Finally, the model does not currently differentiate between correct and incorrect endotracheal tube depth, as bronchial mainstem intubation still results in inflation of both balloons. One possible solution could involve refining the anatomical carina by shortening the current length of the trachea by 1.5-2 inches and replacing the Tee fitting with a Polyvinyl Chloride (PVC) Y-connector. Each branch of the Y-connector would be extended with a short segment of PVC pipe terminating in a balloon to represent the right and left lungs. This setup would allow for correct endotracheal tube placement to inflate both balloons. In contrast, bronchial mainstem intubation would result in inflation of only one balloon, providing immediate and realistic feedback to the learner.

## Conclusions

Our low-cost advanced airway trainer provides EMS personnel and trainees with accessible, realistic practice for emergency airway procedures. At a nominal cost, the customizable design represents a markedly lower-cost alternative to commercial models. Additionally, the customizable design of this task trainer allows flexibility in the selection and use of adjuncts, especially in resource-limited settings. 

We anticipate that implementing this model in educational programs may enhance skill development and confidence, potentially contributing to improved success rates for advanced airway management and better patient outcomes in prehospital care. Further evaluation by clinicians who routinely perform intubations is necessary to determine the model’s effectiveness and realism in simulation-based training.

## Appendices

Appendix A

Google Doc outlining step-by-step instructions for making the model:

https://docs.google.com/document/d/1uZbW6X77pFs1HYkHebI8mI-z1YfeQXcDBshG6rWYqSs/edit?tab=t.0
